# When a partner objectifies other women: Associations with women’s body image, self-esteem, and relationship satisfaction over an eight-month period

**DOI:** 10.1177/17455057261483474

**Published:** 2026-08-22

**Authors:** Eugénie Beaupré-Battisti, Rosalie Lacerte, Camille Lavoie, Geneviève Lavigne, Noémie Carbonneau

**Affiliations:** 1 Department of Psychology, Université du Québec à Trois-Rivières, Trois-Rivières, QC, Canada; 2 Institute of Nutrition and Functional Foods (INAF), Quebec City, QC, Canada; 3 Department of Psychology, Université du Québec à Montréal (UQAM), Montreal, QC, Canada; 4 Research Center of the Montreal Mental Health University Institute (CR-IUSMM), Montreal, QC, Canada

**Keywords:** objectification, body image, relationship satisfaction, self-esteem, longitudinal design

## Abstract

**Background:**

Research indicates that sexual objectification (i.e., treating a person as an object and reducing them to their physical appearance or body parts) within romantic relationships is linked to adverse outcomes such as lower self-esteem and relationship satisfaction. However, no prior research has examined women’s perceptions of their romantic partner’s sexually objectifying behaviors toward other women, nor how these perceptions relate to their own psychological and relational outcomes over time.

**Objectives:**

The present study extends the sexual objectification literature by testing a cross-lagged panel structural equation model (SEM) assessing reciprocal associations between women’s perceived partner objectification of other women and their own body image, self-esteem, and relationship satisfaction.

**Design:**

A two-wave longitudinal design was used.

**Methods:**

A total of 388 French-Canadian women (*M* = 36.2 years, *SD* = 8.6 years, range = 18-66) currently in a romantic relationship completed two self-reported questionnaires eight months apart.

**Results:**

The analyses revealed temporal stability across all constructs, indicating that individual differences were largely stable over time. Nonetheless, women’s perceptions of their partner’s sexually objectifying behaviors toward other women predicted subsequent decreases in self-esteem and relationship satisfaction, but no significant changes in body surveillance. No evidence emerged for reciprocal effects, suggesting that perceived partner objectification predicted later outcomes but was not itself shaped by them.

**Conclusion:**

Overall, these findings suggest that perceiving one’s partner as sexually objectifying other women may undermine women’s psychological and relational well-being, underscoring the importance of integrating objectifying dynamics into couple-focused research and intervention.

## Plain Language Summary

This study examined how women’s perceptions of their romantic partners’ sexually objectifying behaviors toward other women are associated with their own body image, self-esteem, and relationship satisfaction over time. Sexual objectification occurs when individuals are treated as physical objects, valued mainly for their appearance or body parts. Previous research has shown that being personally objectified can be harmful, but little was known about how perceiving a partner objectify others might affect women in romantic relationships. To explore this question, 388 French-Canadian women involved in a romantic relationship completed two surveys, eight months apart. The study used a longitudinal design to examine whether perceived partner objectification was associated with subsequent changes in body image, self-esteem, and relationship satisfaction over time, whether these outcomes were associated with later changes in perceived partner objectification, or both. Findings showed that the variables examined remained relatively stable over the eight-month period. However, perceived partner objectification of other women at baseline was associated with lower self-esteem and relationship satisfaction eight months later, but did not predict significant changes in body image. Results also showed that women’s body image, self-esteem, and relationship satisfaction at baseline did not significantly predict changes in perceived partner objectification over time. Overall, the results suggest that perceiving a partner as sexually objectifying other women may be linked to poorer psychological and relational well-being among women. These findings highlight the importance of considering objectification within romantic relationships, both in research and in efforts aimed at fostering equality and mutual respect between partners.

## 1. Introduction

Interpersonal sexual objectification is a growing area of research. Sexual objectification occurs when a person is treated as an object, reduced to their physical appearance or body parts.^1^ Emerging evidence suggests that such behaviors within romantic relationships may influence interpersonal dynamics, including perceived power, feelings of shame, self-esteem, and overall relationship satisfaction.^2,3^ Sexual objectification, through both interpersonal interactions and the sexualization of women, may reinforce harmful gender stereotypes that normalize violence against women and shape attitudes about acceptable behavior in romantic relationships.^4,5^

According to objectification theory, repeated exposure to objectifying contexts may lead individuals, particularly women, to internalize these evaluations, a process referred to as self-objectification.^1^ In romantic contexts, self-objectification may be triggered not only by societal messages but also by a partner’s objectifying behaviors, which can exacerbate negative outcomes such as body dissatisfaction and lower relationship satisfaction.^6,7^

While previous literature shows that interpersonal objectification within couples can have detrimental effects on women’s well-being and mental health, little is known about the consequences of objectifying behaviors from one’s romantic partner toward other women. Therefore, the present study explores how women’s perceptions of their partners’ sexual objectification of other women relate to their own body image, self-esteem, and relationship satisfaction. Filling this gap will contribute to a better understanding of the consequences of objectifying behaviors, potentially guiding the development of targeted interventions aimed at preventing objectification within relationships.

### 1.1 Objectification theory

Objectification theory posits that women are socialized to internalize the evaluation of their physical appearance, which can shape their self-perceptions and relational experiences.^1^ Objectification entails viewing a person’s body as an object to be observed and evaluated. In other words, it occurs when a person’s entire body, a particular body part, or their sexual features are treated as though they wholly define the individual.^1^ Examples of objectifying behaviors include whistling at someone on the street, visually scrutinizing someone’s body during a conversation, and making sexual or sexist comments.^1,8^ Objectification is closely intertwined with gender identity. While individuals who identify as men can also be affected, objectification appears to have a more pronounced impact on those who identify as women.^9^

A study among young adults revealed that the appearance norms targeting women are significantly more rigid and pervasive than those directed at men.^10^ These norms often promote the illusion that achieving the ideal appearance is both necessary and attainable for women. These rigid norms may be more detrimental to body image than the comparatively more flexible norms typically experienced by men.^10^ According to objectification theory, one explanation for this gender gap is that sexual objectification is deeply embedded in cultures born of patriarchy that emphasize appearance-based norms, values, and ideals for women.^1^

It is noteworthy that gender disparities also emerge in eye-tracking research, suggesting that objectification involves unconscious and automatic cognitive processes. For instance, an eye-tracking study found that men are more likely than women to engage in visual objectification, meaning they scrutinize body parts such as women’s chests and hips.^11^ These results add to a substantial body of literature indicating that women are objectified more frequently and with greater consequences compared to men.^12,13^ Such pressures may influence how women experience interpersonal interactions, including their romantic relationships.^6^ Such findings have important implications for the psychological health of women. Notably, when they feel objectified, (e.g., by others or by society), they may begin to self-objectify and monitor their body’s appearance.^1^

Self-objectification involves viewing and treating oneself as an object to be evaluated based on one’s appearance. It entails perceiving oneself from a third-person perspective rather than from one’s own internal viewpoint.^1^ The focus shifts to physical aspects, such as weight or height, rather than to personal feelings or physical abilities.^14^ Self-objectification can lead to self-surveillance, a persistent self-consciousness characterized by habitually monitoring one’s body appearance.^1^ This self-surveillance encourages individuals to evaluate their appearance by comparing themselves to normative beauty standards and the appearance of their peers.^14^

Self-objectification has been associated with various adverse outcomes that affect overall well-being, including dietary imbalances, sexual dysfunction, depressed mood, disturbances in emotional states, feelings of humiliation and disgust toward oneself, and decreased interest in sexual relationships.^1,8,14^ This process is particularly relevant in romantic contexts, where perceived partner objectification of other women may heighten women’s self-comparisons and body surveillance.^15^

### 1.2 Consequences of objectification

#### 1.2.1 Body image

Body image encompasses individuals’ attitudes, feelings, behaviors, and self-perceptions regarding their physical appearance.^16^ Feeling objectified can lead to several negative consequences for body image, including body shame (i.e., feelings of shame associated with believing that one’s appearance or appearance-related behaviors do not meet personal and cultural standards), appearance anxiety (i.e., anxiety specifically related to negative self-evaluation and others’ judgements of one’s physical appearance), and a disruption in the connection between the mind and internal bodily sensations, such as hunger and thirst.^8,17–19^ In the present study, we examined perceived partner objectification of other women in relation to the specific body image facet of body surveillance. Body surveillance involves persistent self-monitoring to meet external appearance standards.^1^ We posit that body surveillance may be heightened when women perceive their romantic partner as objectifying other women, as this experience may promote self-objectification and appearance-based self-evaluation.^7^

#### 1.2.2 Self-esteem

Self-esteem refers to a person’s evaluation of, or attitude toward, themselves.^20^ Recent studies show that sexual objectifying experiences and self-objectification are correlated with lower body esteem, lower global self-esteem, and higher symptoms of anxiety.^21,22^ Some research has shown that frequent encounters with body objectification (e.g., pressures for thinness, appearance commentary) are associated with lower self-esteem in American adult women.^23,24^ Another study demonstrated that greater self-surveillance predicted lower self-esteem, with this relationship being fully mediated by body shame and appearance anxiety.^25^ In fact, low self-esteem has been shown to be a predisposing factor for the internalization of thinness ideals.^26^ However, most of the previously cited research focused on individuals in isolation and on general feelings of self-objectification and being objectified. Research remains limited on self-esteem and objectification within specific relationships, notably within romantic relationships. When women perceive their partner to objectify other women, they may internalize such appearance-based standards, which may contribute to self-objectification and more negative self-evaluations, with downstream implications for self-esteem, as suggested by broader objectification research.^15,27^

#### 1.2.3 Romantic relationships

Recent research examining romantic relationships through the lens of objectification theory suggests that these relationships constitute an important context in which to study objectification, as partners can significantly influence each other’s concerns about physical appearance and relationship satisfaction, either exacerbating or alleviating them.^28,29^ Within couples, objectification can manifest through various behaviors, such as comparing one’s partner’s appearance to that of others or making frequent comments about a partner’s physical appearance.^30^

Objectification within couples can harm the relationship satisfaction of both the person who is objectified and the one who engages in objectification.^31^ A recent study found that the more individuals objectify their partner, the lower their relationship satisfaction and sexual satisfaction.^31^ The same study also showed that being the target of partner objectification is associated with greater body dissatisfaction and lower sexual satisfaction.^31^ Furthermore, the more individuals focus on their partner’s physical appearance over their personality and competence, the more likely the partner is to self-objectify.^32^ In sum, previous research suggests that objectification in romantic relationships is associated with lower relationship quality. Accordingly, perceiving one’s partner to objectify other women may undermine relationship satisfaction by fostering insecurity, social comparison, and emotional distance between partners.^3,9^

### 1.3 The present study

Building on the literature on objectification within romantic relationships, the present study focuses on three key outcomes through which perceived partner objectification of other women may influence women’s experiences: body surveillance, self-esteem, and relationship satisfaction. These outcomes were selected because they reflect distinct but interconnected dimensions of women’s intrapersonal and relational functioning.

The few existing studies on objectification within romantic relationships have primarily focused on how partners objectify each other.^31,33,34^ As a result, women’s perceptions of their partners’ sexually objectifying behaviors toward other women remain understudied. Little is known about whether and how such behaviors relate to women’s body image, self-esteem, and relationship satisfaction. Addressing this gap could enhance understanding of these associations and support the development of targeted interventions to promote women’s psychological health and well-being. Furthermore, most objectification research has focused on heterosexual couples. The present study seeks to broaden the scope by including participants in both heterosexual and same-gender relationships.

### 1.4 Objectives and hypotheses

The aim of this study was to examine women’s perceptions of their partners’ sexually objectifying behaviors toward other women in relation to body image, self-esteem, and relationship satisfaction across an eight-month period, using a fully reciprocal cross-lagged panel model.

Specifically, we examined whether women’s perceived partner objectification of other women was related to changes in their body image (i.e., body surveillance), self-esteem, and relationship satisfaction over time. We also investigated whether changes in these outcome variables were linked to variations in women’s perceived partner objectification of other women. We hypothesized that increases in perceived partner objectification of other women would predict greater self-conscious body surveillance, and lower self-esteem and relationship satisfaction over time, whereas the reverse associations were expected to be non-significant.

## 2. Methods

### 2.1 Participants and procedure

To take part in the study, participants had to identify as women, be aged 18 or older, have been in a romantic relationship with the same partner for at least six months, and understand French well. The reporting of this study is in accordance with the STROBE statement.^35^ Participants were excluded if they did not meet one or more of these inclusion criteria. Of the 1,072 participants who started the questionnaire at baseline (Time 1), 852 met the inclusion criteria and completed it in full. They were invited to complete a similar questionnaire eight months later (Time 2). At Time 2, 465 participants began the survey but eight were excluded because they were no longer in a relationship with the same partner. Additionally, some participants did not complete the questionnaire adequately, resulting in a final sample of 388 participants who completed both Time 1 and Time 2. Based on model complexity, SEM recommendations indicated a minimum of 360 participants,^36–38^ which was exceeded by the final sample (N = 388). Power analysis (α = .05, power = .80, effect size = 0.30)^39^ further indicated a required minimum of 177 participants, confirming sufficient statistical power.

The participants were self-identified women aged between 18 and 66 years (*M* = 36.2, *SD* = 8.6). A total of 383 participants identified as cisgender women, while five identified either as gender fluid or nonbinary. The majority (98.5%) identified as White, and 69.6% held a university degree. Gross annual family income exceeded $90,000 CAD for 58% of participants, and 99.2% reported French as their native language.

Most participants were in a relationship with a man (96.9%), while a minority were either in a relationship with a woman (2.6%) or did not specify their partner’s gender identity (0.5%). The length of relationships at Time 1 ranged from six months to 32.4 years (*M* = 9.0 years, *SD* = 7.2 years). Regarding relationship status, 20.6% of participants reported being married and living with their partner, 65.4% were cohabiting without being married, and 14.0% were neither married nor cohabiting. One participant did not report her relationship status.

This study was conducted over eight months and included two data collections time points: Time 1 in June 2022 and Time 2 in February 2023. The project was approved by the Research Ethics Committee of Université du Québec à Trois-Rivières (Approval No. CERPPE-22-06-07.05) on May 10, 2022. Participants were recruited online through a paid Facebook advertisement. The ad contained a message explaining that the questionnaire would take approximately 25 minutes to complete and included a hyperlink directing participants to the study’s information and consent letter. Informed consent was documented electronically, with participants explicitly approving or declining their participation by checking the appropriate box on the online consent form. After giving informed consent, participants completed the questionnaire, which included the study measures. At Time 1, participants could optionally provide their email address to enter a draw for three $50 CAD prizes and/or to be contacted for Time 2. At Time 2, each participant who completed the questionnaire received a $10 CAD compensation. All measures were completed online.

### 2.2 Measures

#### 2.2.1. Demographics

Participants first completed a sociodemographic questionnaire including questions about their age, gender identity, main current occupation, highest level of completed education, first language, ethnicity, and family income. They were also asked to report on characteristics of their romantic relationship, such as relationship length, partner’s gender identity, and relationship status (e.g., married, cohabiting). Participants then completed the measures assessing the study’s variables of interest. Any measure originally available only in English was translated into French using a back-translation procedure.^40^ Reliability analyses in the present sample indicated good internal consistency for all scales (Cronbach’s alpha ranged from 0.85 to 0.92 across Time 1 and Time 2).

#### 2.2.2. Perceived partner objectification of other women

The Interpersonal Sexual Objectification Scale^41^ was used to assess perceptions of one’s romantic partner’s sexually objectifying behaviors toward other women. The scale comprises 15 items rated on a five-point Likert scale ranging from 1 (Never) to 5 (Almost always). A sample item is: “To the best of your knowledge, to what extent does your partner tend to stare at a woman’s chest while talking to her?” A total score was calculated by averaging responses across all items. Cronbach’s alpha was 0.85 at both Time 1 and Time 2.

#### 2.2.3. Body surveillance

The 8-item body surveillance subscale of the Objectified Body Consciousness Scale^19^ was used to assess self-conscious body surveillance. Items are rated on a Likert scale ranging from 1 (Strongly disagree) to 7 (Strongly agree). A sample item is: “During the day, I often think about my appearance.”) A total score was calculated by averaging responses across the 8 items. Cronbach’s alpha for this subscale was from 0.86 Time 1 and 0.85 at Time 2.

#### 2.2.4. Self-esteem

The Self-Esteem Scale^42^ was used to assess participants’ overall self-esteem. The scale comprises 10 items, rated on a four-point Likert scale ranging from 1 (Strongly disagree) to 4 (Strongly agree). A sample item is: “I have a positive attitude toward myself.” A total score was calculated by averaging responses across all items. Cronbach’s alpha was 0.89 at both Time 1 and Time 2.

#### 2.2.5. Relationship satisfaction

The Relationship Assessment Scale^43^ was used to assess general satisfaction with one’s romantic relationship. The scale comprises seven items rated on a five-point Likert scale ranging from 1 (e.g., not satisfied) to 5 (e.g., very satisfied). A sample item is: “In general, how satisfied are you with your relationship?”. A total score was calculated by averaging responses across all items. Cronbach’s alpha was 0.90 at Time 1 and 0.92 at Time 2.

### 2.3 Statistical analysis

The means, standard deviations, and correlations of the model’s variables are presented in Table 1. To explore the study hypotheses, a structural equation model was tested using AMOS 29.^44^ The goodness of fit of the model was assessed using the following fit indices: Normed Fit Index (NFI), Goodness of Fit Index (GFI), Comparative Fit Index (CFI), Standardized Root Mean Square (SRMR), and Root Mean Square Error of Approximation (RMSEA). The cut-off values for these fit indices were NFI > 0.90, GFI > 0.90, CFI > 0.90, SRMR < 0.10, and RMSEA < 0.05.^45^

**Table 1.** Means, standard deviations and correlations among the study variables.

​	Mean	SD	1	2	3	4	5	6	7	8
1. Perceived Partner Objectification of Other Women (T1)	1.326	.312	1	.085	−.038	−.274***	.808***	−.117*	−.103*	−.295***
2. Body Surveillance (T1)	4.185	1.121		1	−.386***	−.212**	.061	.803***	−.345***	−.191***
3. Self-Esteem (T1)	3.212	.554			1	.295***	.017	−.358***	.803***	.225***
4. Relationship Satisfaction (T1)	4.249	.677				1	-.236***	−.203***	.304***	.843***
5. Perceived Partner Objectification of Other Women (T2)	1.306	.317					1	.057	−.048	−.310***
6. Body Surveillance (T2)	4.108	1.114						1	−.406***	−.192***
7. Self-Esteem (T2)	3.195	.533							1	.270***
8. Relationship Satisfaction (T2)	4.201	.730								1

*Note. N* = 388 women. Perceived partner objectification of other women and relationship satisfaction are scored on scales ranging from 1 to 5; body surveillance is scored on a scale ranging from 1 to 7; self-esteem is scored on a scale ranging from 1 to 4.

**Figure 1.**
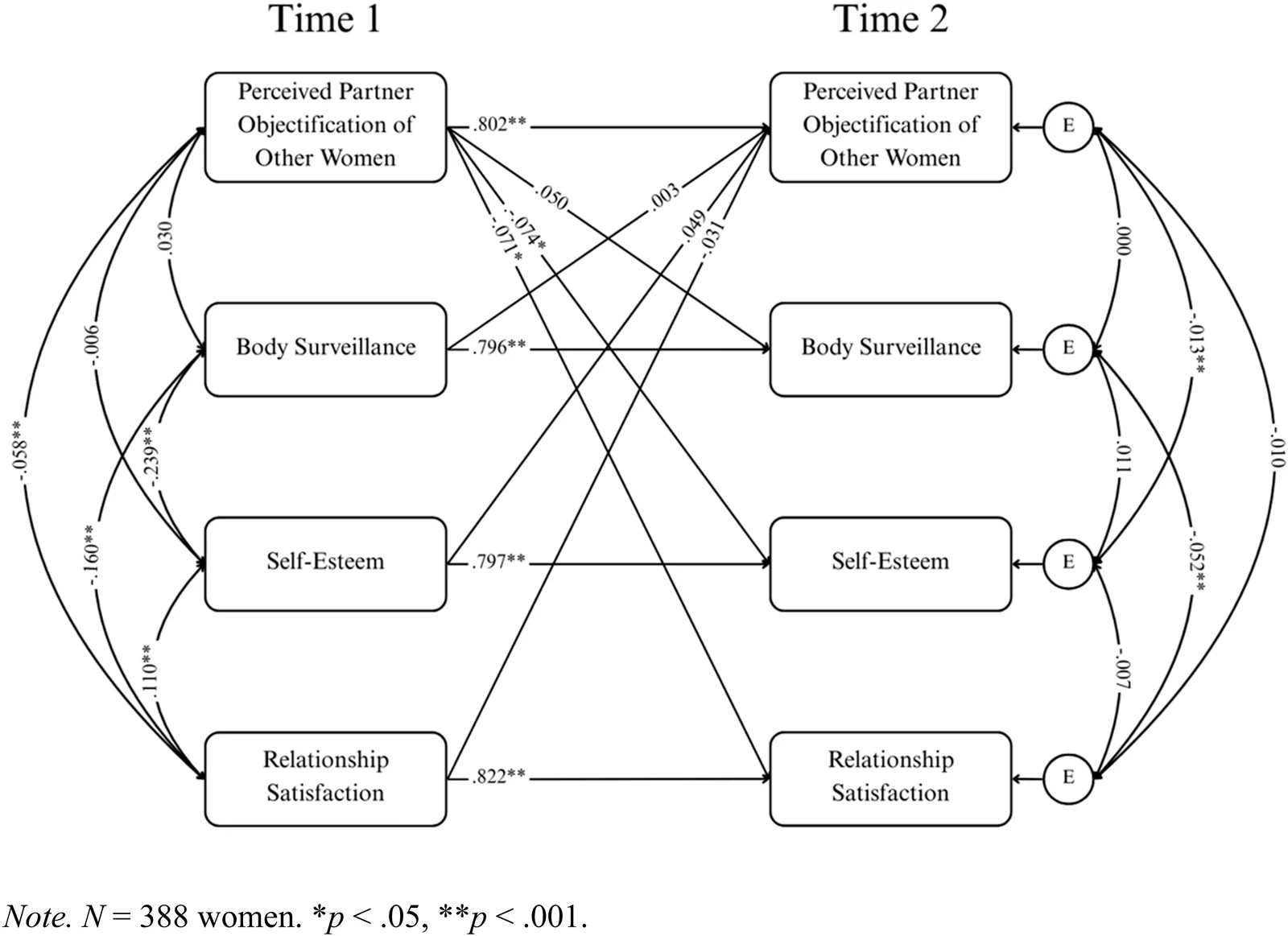
Results of the fully reciprocal cross-lagged panel model.

The model tested in the present study was composed of eight variables (see Figure 1): four exogenous variables (i.e., perceived partner objectification of other women, body surveillance, self-esteem, and relationship satisfaction at Time 1) and four endogenous variables (i.e., the same variables at Time 2). To test the hypotheses of the present study, a fully reciprocal cross-lagged panel model was specified with a total of 10 paths: autoregressive paths between each variable at Time 1 and its equivalent at Time 2 (to account for temporal stability), paths from perceived partner objectification of other women at Time 1 to each outcome variable at Time 2 (i.e., body surveillance, self-esteem, and relationship satisfaction), as well as paths from each outcome variable at Time 1 (i.e., body surveillance, self-esteem, and relationship satisfaction) to perceived partner objectification at Time 2. This design allowed for the estimation of bidirectional effects.

## 3. Results

### 3.1 Structural equations modeling analyses

This fully reciprocal cross-lagged panel model resulted in adequate fit indices: χ^2^ (*df* = 6, *N* = 388) = 8.30, *p* = .217, NFI = 1.00, CFI = 1.00, SRMR = .02, and RMSEA = .031 [.000; .078]. The standardized solution is presented in Figure 1.

Each variable at Time 1 was strongly and positively associated with its equivalent at Time 2 (βs ranging from 0.80 to 0.82, all *p*s < .001), suggesting that the constructs were highly stable over time. Although a substantial proportion of the variance of each Time 2 outcome was explained by its equivalent at Time 1, additional variance was accounted for by perceived partner objectification of other women at Time 1. More specifically, perceived partner objectification at Time 1 predicted a small, albeit not significant, increase in body surveillance (β = .05, *p* = .10), and significant decreases in self-esteem (β = -.07, *p* < .05) and relationship satisfaction (β = -.07, *p* < .05) over the eight-month period. In contrast, the reverse effects were weak or non-significant: Time 1 outcome variables did not reliably predict changes in perceived partner objectification of other women at T2 (body surveillance: β = .00, *p* = .922; self-esteem: β = .05, *p* = .135; relationship satisfaction: β = -.03, *p* = .341). The model was also re-estimated while controlling for relationship length, and the patterns of results remained unchanged.

## 4. Discussion

Although objectification has been previously linked to higher appearance anxiety and body shame, as well as lower self-esteem,^1,8,19,46^ the present study was the first to examined how women’s perceptions of their romantic partner’s sexually objectifying behaviors toward *other women* relate to their own body image, self-esteem, and relationship satisfaction over time. We hypothesized that perceived partner objectification of other women would predict, over time, a more negative body image (i.e., higher body surveillance), lower self-esteem, and reduced relationship satisfaction in women. Overall, the results of the present study supported most of these hypotheses.

Results of a fully reciprocal cross-lagged panel model showed that perceived partner objectification of other women at baseline predict lower self-esteem eight months later. Based on previous research, this association might be explained by the internalization of objectification and appearance-contingent self-worth.^47^ Indeed, having a partner who displays objectifying behaviors toward women could lead a person to base her self-worth on physical appearance.^47^ Another hypothesis is that exposure to objectifying behaviors could diminish one’s self-esteem and confidence by reinforcing the belief that objectified women are less intelligent and competent. Individuals who perceive that their partner frequently engages in sexually objectifying behaviors may begin to question their own desirability, wondering whether they are interesting or attractive for their partner. Future studies should explore whether the internalization of competency beliefs can mediate the association between perceived partner objectification of other women and lower self-esteem.

In the present study, higher perceived partner objectification of other women at baseline was associated with lower relationship satisfaction eight months later. This finding is consistent with previous studies showing that self-objectification and objectification of one’s partner are associated with lower relationship satisfaction.^3,9^ Some individuals may perceive their partner sexually objectifying other women as a form of infidelity, which can induce feelings of jealousy, undermine emotional security, and lead them to question their partner’s loyalty. Repeated manifestations of sexual objectification by one’s partner toward other women may reflect a mismatch in core values (such as respect or commitment), ultimately compromising relationship satisfaction and cohesion.

Contrary to our hypotheses, perceived partner objectification of other women did not predict significant changes in women’s body surveillance over time. This finding suggests that exposure to objectification directed at others may not be internalized in the same way as direct experiences of objectification. It is possible that some women attribute these behaviors to their partner’s personality or habitual tendencies, thereby reducing their relevance for body-related self-evaluations. It is also plausible that women who feel that their body image is threatened by their partner’s objectifying behaviors may be more likely to leave such relationships, whereas those who remain with a partner who engages in these behaviors may be less affected by them. The absence of a statistically significant association for body surveillance may reflect meaningful variability in how partner objectification of other women is understood and integrated into self-concept, highlighting the need for further research on theses processes.

An interesting avenue for future research would be to examine intrapersonal and interpersonal factors that may moderate the associations between perceived partner objectification and body image, self-esteem, and relationship satisfaction. For example, women who endorse more traditional gender norms or who idealize their partner may interpret objectifying behaviors as socially normative expressions of attraction, thereby minimizing their impact on their own body image. Thresholds for perceiving partner behaviors as objectifying may also vary across women; some may construe them as harmless, whereas others may interpret them as disrespectful or invalidating, leading to different emotional consequences.

The fully reciprocal cross-lagged model that was tested in the present study allowed us to examine (a) whether perceived partner objectification of other women predicts changes in body image, self-esteem, and relationship satisfaction, (b) whether changes in body image, self-esteem and relationship satisfaction predict changes in perceived partner objectification, (c) or both. While perceived partner objectification was found to predict significant decreases in both self-esteem and relationship satisfaction over an eight-month period, the reverse associations were not found to be significant. It might have been reasonable to expect that a person with low self-esteem and/or who is unhappy in her relationship would be more likely to perceive that her partner objectifies other women, but it was not observed. Rather, the results support the hypothesis that perceptions of a partner’s objectifying behaviors may come to affect one’s self-view and evaluation of the relationship over time. Regarding body image, it did not significantly predict changes in perceived partner objectification over time, nor was the reverse association significant. A study spanning a period longer than eight months may have yielded different patterns of results, which remains to be empirically tested.

### 4.1 Strengths and limitations

This is an original study that uniquely examines women’s perceptions of their partner’s objectification of other women, addressing a gap in the existing literature. Our large sample size allowed for a diverse range of responses. Importantly, this longitudinal design made it possible to observe changes over time, providing valuable insights into temporal dynamics. The longitudinal approach also allowed for stronger inferences regarding the directionality of associations, helping to clarify how the associations between perceptions of partner objectification, body image, self-esteem, and relationship satisfaction unfold over time. We show that women’s perceptions of their partner’s objectification of other women are an important variable to consider, as these perceptions may affect both their self-esteem and relationship satisfaction over time. These findings suggest that the reach of objectification is not limited to *direct* experiences but also extends to *indirect* observations, thereby broadening the scope of research on objectification. From a clinical perspective, these results underscore the value of addressing objectifying behaviors in couple therapy, whether toward one’s partner or other individuals. Interventions could also help partners recognize and reflect on how these behaviors are perceived and interpreted, by fostering open communication, promoting alignment in relational values, and increasing partners’ awareness of the potential impact of these dynamics on partners’ well-being.

Some limitations of the present study must be acknowledged. First, our sample is limited by the underrepresentation of women in same-sex relationships, despite our best intentions to include sexual minorities. Most studies on objectification focus on heterosexual individuals, thus excluding those from sexual minorities. Among the few studies that explored objectification within romantic relationships, all were conducted exclusively among heterosexual couples, which hinders our understanding of objectification’s psychological impacts within sexual minority couples. The generalizability of the findings is therefore constrained by the predominance of heterosexual participants. In addition, the relative homogeneity of the sample, composed primarily of French-Canadian and highly educated participants, further limits the broader applicability of the findings. Future studies should aim to include participants with more diverse sexual orientations, gender identities, cultural backgrounds, and socioeconomic statuses to enhance the external validity of these findings. Expanding recruitment to include a wider range of relationship types would further strengthen generalizability. Another limitation of the current study is its reliance on self-reported data from a single partner, which captures only a partial view of objectification processes within the relationship. Future research could address this limitation by adopting a dyadic design in which both partners report their own objectifying behaviors, as well as those they perceive in their partner. Such a design would provide a more comprehensive understanding of how objectification is both enacted and perceived within romantic dynamics. Additionally, future studies should investigate whether participants perceive objectifying behaviors from their partner as being directed exclusively toward other women or whether they also perceive these behaviors as being directed toward themselves. Investigating potential moderating factors, such as individual tendencies to objectify others or endorsement of certain social norms and values, could further clarify when and for whom perceived objectification is associated with relational and psychological outcomes.

## 5. Conclusion

In conclusion, the present study supports and extends objectification theory,^1^ stressing the importance of the perceptions of one’s partner’s sexually objectifying behaviors toward other women for women’s self-esteem and relationship satisfaction. Our study goes beyond previous research that focused on the objectification experienced within the couple itself. To our knowledge, this is the first study to examine the perceptions of partner objectification of other women. We found that perceived partner objectification of other women prospectively predicts decreases in self-esteem and relationship satisfaction over an eight-month period. These results contribute to a growing body of knowledge in the field of objectification, while also offering new avenues of research related to women’s perceptions of objectification. Importantly, these results may have implications for couple counselling and psychotherapy, particularly for the development of new intervention avenues targeting objectifying behaviors.

## Data Availability

The first author of this paper has full access to the data reported in the manuscript. All data used in this study will be made available upon reasonable request.*

## References

[1] Fredrickson BL Roberts TA . Objectification Theory: Toward Understanding Women's Lived Experiences and Mental Health Risks. Psychol Women Q 1997; 21: 173–206. 10.1111/j.1471-6402.1997.tb00108.x

[2] Pecini C Di Bernardo GA Tallone B , et al. Who Is in Charge? Partner-Sexual Objectification, Personal Power, and Relationship Satisfaction in Heterosexual Women. Psychol Women Q 2024; 1: 264–276. 10.1177/03616843241307015

[3] Ramsey LR Marotta JA Hoyt T . Sexualized, objectified, but not satisfied: Enjoying sexualization relates to lower relationship satisfaction through perceived partner-objectification. J Soc Pers Relat 2017; 34: 258–278. 10.1177/0265407516631157

[4] Wright PJ Tokunaga RS . Men’s Objectifying Media Consumption, Objectification of Women, and Attitudes Supportive of Violence Against Women. Arch Sex Behav 2016; 45: 955–964. 10.1007/s10508-015-0644-8 26585169

[5] Pecini C Ruzzante D Valtorta RR , et al. Why Might Women Justify Dating Violence? The Role of Men’s Sexual Objectification of Their Romantic Partners Within Heterosexual Relationships. J Interpers Violence 2023; 38: 10664–10685. 10.1177/08862605231175515 37227007

[6] Brock RL Gervais SJ Checkalski OR , et al. A Multidimensional Measure of Sexual Objectification in Intimate Relationships: The Inventory of Partner Sexual Objectification (IPSO). Psychological Assessment 2025; 37: 720–734. 10.1037/pas0001411 40742767

[7] Read K Kılıç D Kahalon R , et al. The dual lens of objectification: Perceived objectification, male partners’ reported objectification, and women's detrimental sexual outcomes. J Soc Pers Relat 2025; 42: 717–741. 10.1177/02654075241304802

[8] Calogero RM . Objectification theory, self-objectification, and body image. In: Cash T (ed). Encyclopedia of body image and human appearance. Academic Press, 2012, pp. 574–580.

[9] Zurbriggen E Ramsey L Jaworski B . Self- and Partner-objectification in Romantic Relationships: Associations with Media Consumption and Relationship Satisfaction. Sex Roles 2011; 64: 449–462. 10.1007/s11199-011-9933-4 21475650 PMC3062032

[10] Buote VM Wilson AE Strahan EJ , et al. Setting the bar: Divergent sociocultural norms for women's and men's ideal appearance in real-world contexts. Body Image 2011; 8: 322–334. 10.1016/j.bodyim.2011.06.002 21775228

[11] Gervais SJ Holland AM Dodd MD . My Eyes Are Up Here: The Nature of the Objectifying Gaze Toward Women. Sex Roles 2013; 69: 557–570. 10.1007/s11199-013-0316-x

[12] Heflick NA Goldenberg JL Cooper DP , et al. From women to objects: Appearance focus, target gender, and perceptions of warmth, morality and competence. J Exp Soc Psychol 2011; 47: 572–581. 10.1016/j.jesp.2010.12.020

[13] Vaes J Paladino P Puvia E . Are sexualized women complete human beings? Why men and women dehumanize sexually objectified women. Eur J Soc Psychol 2011; 41: 774–785. 10.1002/ejsp.824

[14] Wollast R De Wilde M Bernard P , et al. Percevoir son corps à travers le regard d’autrui: une revue de la littérature sur l’auto-objectification. L’Année psychologique 2020; 120: 321–347. 10.3917/anpsy1.203.0321

[15] Strelan P Hargreaves D . Women Who Objectify Other Women: The Vicious Circle of Objectification? Sex Roles 2005; 52: 707–712. 10.1007/s11199-005-3737-3

[16] Van den Brink F Vollmann M Smeets MAM , et al. Relationships between body image, sexual satisfaction, and relationship quality in romantic couples. J Fam Psychol 2018; 32: 466–474. 10.1037/fam0000407 29517245

[17] Tiggemann M Lynch JE . Body image across the life span in adult women: The role of self-objectification. Dev Psych 2001; 37: 243–253. 10.1037/0012-1649.37.2.243 11269392

[18] Trevor AH Flora DB Palyo SA , et al. Development and Examination of the Social Appearance Anxiety Scale. Assessment 2008; 15: 48–59. 10.1177/1073191107306673 18258731

[19] McKinley NM Hyde JS . The objectified body consciousness scale: Development and validation. Psychology of Women Quarterly 1996; 20: 181–215. 10.1111/j.1471-6402.1996.tb00467.x

[20] James W . The principles of psychology. Henry Holt and Company, 1890.

[21] Behera N Khuntia S . Self-esteem, Self-objectification, Appearance Anxiety, Resilience, and Gender: Testing a Moderated Mediational Analysis. Open Psychol J 2025; 18: e18743501347628. 10.2174/0118743501347628250114054620

[22] Sherman AM Tran S Sy J . Objectification and body esteem: age group patterns in women's psychological functioning. Aging Ment Health 2024; 28: 706–716. 20231102. 10.1080/13607863.2023.2273338 37916646

[23] Herbozo S Thompson JK . Appearance-related commentary, body image, and self-esteem: Does the distress associated with the commentary matter? Body Image 2006; 3: 255–262. 10.1016/j.bodyim.2006.04.001 18089228

[24] Phan T Tylka TL Annual Convention of the American Psychological A . Exploring a model and moderators of disordered eating with Asian American college women. J Couns Psychol 2006; 53: 36–47. 10.1037/0022-0167.53.1.36

[25] Choma BL Visser BA Pozzebon JA , et al. Self-Objectification, Self-Esteem, and Gender: Testing a Moderated Mediation Model. Sex Roles 2010; 63: 645–656. 10.1007/s11199-010-9829-8

[26] Striegel-Moore RH Cachelin FM . Body image concerns and disordered eating in adolescent girls: Risk and protective factors. Beyond appearance: A new look at adolescent girls. American Psychological Association, 1999, pp. 85–108.

[27] Kranz D . When perceived partner-objectification contaminates women's self-esteem: On the mediating effects of self-objectification and body appreciation. Curr Psychol 2025; 44: 15668–15680. 10.1007/s12144-025-08211-1

[28] Meltzer AL McNulty JK . Tell me I’m sexy. . . and otherwise valuable’: Body valuation and relationship satisfaction. Pers Relatsh 2014; 21: 68–87. 10.1111/pere.12018 24683309 PMC3964620

[29] Overstreet N Quinn D Marsh K . Objectification in Virtual Romantic Contexts: Perceived Discrepancies Between Self and Partner Ideals Differentially Affect Body Consciousness in Women and Men. Sex Roles 2015; 73: 442–452. 10.1007/s11199-015-0533-6 26594085 PMC4652648

[30] Mahar EA Webster GD Markey PM . Partner-objectification in romantic relationships: A dyadic approach. Pers Relatsh 2020; 27: 4–26. 10.1111/pere.12303

[31] Sáez G Riemer AR Brock RL , et al. Objectification in Heterosexual Romantic Relationships: Examining Relationship Satisfaction of Female Objectification Recipients and Male Objectifying Perpetrators. Sex Roles 2019; 81: 370–384. 10.1007/s11199-018-0990-9

[32] Strelan P Pagoudis S . Birds of a Feather Flock Together: The Interpersonal Process of Objectification within Intimate Heterosexual Relationships. Sex Roles 2018; 79: 72–82. 10.1007/s11199-017-0851-y

[33] Pecini C Di Bernardo GA Crapolicchio E , et al. Stop looking at me! associations between men’s partner-objectification and women’s self-objectification, body shame and life satisfaction in romantic relationships. J Community Appl Soc Psychol 2022; 32: 1047–1060. 10.1002/casp.2627

[34] Ramsey LR Hoyt T . The Object of Desire: How Being Objectified Creates Sexual Pressure for Women in Heterosexual Relationships. Psychol Women Q 2015; 39: 151–170. 10.1177/0361684314544679

[35] Initiative S Erik von E Altman DG , et al. The Strengthening the Reporting of Observational Studies in Epidemiology (STROBE) Statement: guidelines for reporting observational studies. WHO Bulletin of the WHO 2007; 85: 867–872. 10.2471/BLT.07.045120 PMC2636253 18038077

[36] Bonneville-Roussy A Fenouillet F Morvan Y . Introduction aux analyses par équations structurelles: applications avec Mplus en psychologie et sciences sociale. Dunod, 2022.

[37] Kline RB . Principles and practice of structural equation modeling. Fourth edition ed. The Guilford Press, 2016.

[38] Wang J Wang X . Structural equation modeling: applications using Mplus. Second edition. Wiley, 2020.

[39] Soper DS . Structural equation model sample size calculator [online calculator]. 2025. https://www.danielsoper.com/statcalc (accessed November 6, 2025).

[40] Vallerand RJ . Vers une méthodologie de validation trans-culturelle de questionnaires psychologiques: Implications pour la recherche en langue française. Can Psychol 1989; 30: 662–680. 10.1037/h0079856 6850979

[41] Kozee HB Tylka TL Augustus-Horvath CL , et al. Development and Psychometric Evaluation of the Interpersonal Sexual Objectification Scale. Psychol Women Q 2007; 31: 176–189. 10.1111/j.1471-6402.2007.00351.x

[42] Rosenberg M . Society and the adolescent self-image. Rev ed. Wesleyan University Press, 1989.

[43] Hendrick SS Dicke A Hendrick C . The Relationship Assessment Scale. J Soc Pers Relat 1998; 15: 137–142. 10.1177/0265407598151009

[44] Arbuckle JL . IBM SPSS AMOS 29 user’s guide. IBM Corporation, 2022.

[45] Hair JF Anderson RE Tatham RL , et al. Multivariate analysis of variance. Multivariate data analysis. 4th ed. Macmillan Publishing Company, 1992, pp. 326–386.

[46] Myers TA Crowther JH . Is self-objectification related to interoceptive awareness? An examination of potential mediating pathways to disordered eating attitudes. Psychol Women Q 2008; 32: 172–180. 10.1111/j.1471-6402.2008.00421.x

[47] Adams KE Tyler JM Calogero R , et al. Exploring the relationship between appearance-contingent self-worth and self-esteem: The roles of self-objectification and appearance anxiety. Body Image 2017; 23: 176–182. 10.1016/j.bodyim.2017.10.004 29055772

